# Aerobic exercise impacts the anterior cingulate cortex in adolescents with subthreshold mood syndromes: a randomized controlled trial study

**DOI:** 10.1038/s41398-020-0840-8

**Published:** 2020-05-18

**Authors:** Kangguang Lin, Brendon Stubbs, Wenjin Zou, Wenjing Zheng, Weicong Lu, Yanling Gao, Kun Chen, Shengli Wang, Jie Liu, Yanxiong Huang, Lijie Guan, Mabel Ngai Kiu Wong, Runhua Wang, Bess Yin-Hung Lam, Guiyun Xu

**Affiliations:** 1 Department of Affective Disorders, The Affiliated Brain Hospital of Guangzhou Medical University (Guangzhou Huiai Hospital),Guangzhou Medical University, Guangzhou, China; 2 Academician workstation of Mood and Brain Sciences, Guangzhou Medical University, Guangzhou, China; 3 Department of Psychological Medicine, Institute of Psychiatry, Psychology, and Neuroscience, King’s College London, London, UK; 4 Department of Radiology, The Affiliated Hospital of Guangzhou Medical University (GuangzhouHuiai Hospital), Guangzhou Medical University, Guangzhou, China; 5 Yuanxuan Middle School, Huadu district, Guangzhou, China; 6 Department of Rehabilitation Sciences, The Hong Kong Polytechnic University, Hung Hom, Hong Kong

**Keywords:** Neuroscience, Predictive markers

## Abstract

Aerobic exercise is effective in alleviating mood symptoms while the mechanism is poorly understood. There are limited clinical trials that investigated the effect of exercise on the anterior cingulate cortex (ACC), a key brain region involved in mood regulations, in adolescents with subthreshold mood syndromes. This randomized controlled trial (RCT) of aerobic exercise was undertaken in a middle school in Guangzhou, China. Participants were adolescents aged 12–14 with subthreshold mood syndromes including depressive and manic symptoms and were randomly assigned to an aerobic exercise intervention or a psychoeducation control group. Participants in the exercise group received moderate-intensity exercise intervention, consisting of 30 mins running, 4 days per week for 3 months. The primary outcome in this study was structural changes in the ACC from baseline to post intervention. The trial was registered with ClinicalTrial.gov (NCT03300778). Of 56 participants who met the criteria for subthreshold mood syndromes, 39 (41.03% males) had complete MRI data, with 20 and 19 subjects in the exercise and control group, respectively. At baseline, demographic information (e.g., age and sex), clinical symptoms, and the gray matter volume and cortical thickness of ACC were matched between the two groups. After 12 weeks of treatment, participants in the exercise group displayed increased gray matter volume of the left rostral ACC (F_1,30_ = 5.73, *p* = 0.02) and increased cortical thickness of the right rostral ACC (F_1,30_ = 7.83, *p* = 0.01) when compared with the control group. No significant differences were found for caudal ACC cortical thickness and gray matter volume. Our data demonstrate that 12-week, moderate-intensity aerobic exercise can induce structural changes in the rostral ACC in adolescents with subthreshold mood syndromes.

## Introduction

At least 50% of mental health symptoms and disorders develop in youth and adolescence^1^. The burden of poor mental health and wellbeing remains substantial during this key time of transition^2^. Early intervention during adolescence is key in order to effectively treat the mental health symptoms and prevent any longer term impact during later life^3^.

Mood disorders are a leading cause of mental health burden in adolescence^3^. A number of different treatment approaches have been proposed such as antidepressant medication^4^, psychological therapy^5^, and psychoeducation^6^. However, antidepressant medication may have side effects^7^ and possibly be related increased risk of suicide attempts^8^. Psychological therapy does not work for all adolescents^9^. In addition, there is increasing recognition that mood disorders in adolescence often co-occur with physical health comorbidity such as obesity and diabetes^10–12^ which medication and psychological therapy do not address. Moreover, mood disorders are often associated with impaired cognitive capabilities, which can impede adolescents functioning during this critical time and treatment options are limited^10,12^. Furthermore, adolescents who presented subthreshold depression already displayed abnormalities in the white matter tracts connecting the corpus callosum to the anterior cingulate cortex (ACC), which was associated with higher risk for developing depressive disorder^13^.

Recently, there has been a dramatic rise in interest for the potential for physical exercise to prevent and manage mood disorders in adolescence^14–17^. Physical exercise has been established to have a key role in promoting positive physical health in adolescence such as reduced risk of physical comorbidities^18^. In addition, robust evidence indicates that exercise can improve neurocognition, academic performance, and induce structural changes in the adolescence brain^19,20^. Recently, some systematic review have suggested that exercise can improve mental health symptoms in adolescence^21,22^ with subthreshold and established mood disorders.

Although some progress has been made demonstrating the efficacy of exercise, limited clinical trials have explored the potential underlying mechanisms, particularly in adolescence^23^. A few clinical trials in older adults with or without cognitive impairment showed that ACC is a potential target to be changed by exercise^24^. The ACC serves as a hub for global neural network dynamics underlying an array of functions, e.g., mood regulations, cognitive, and social function^25,26^. Aberrant development of the ACC in adolescents has been implicated in mood disorders including depression and bipolar disorder^27,28^. However, few randomized controlled trial (RCT) studies by far have investigated the exercise effects on adolescents with subthreshold mood symptoms, a high-risk population in developing mood disorders^1^.

Given the above considerations, the aim of this study was to investigate the effects of 12-week moderate-intensity aerobic exercise on the ACC structure. We hypothesized that the aerobic exercise could increase gray matter volume and cortical thickness of the ACC.

## Methods

### Study design and participants

This exercise study is a prospective, two-arm, parallel-group, randomized, controlled trial (NCT03300778). Participants were recruited from a middle school in Guangzhou, China. The study was approved by the IRB of The Affiliated Brain Hospital of Guangzhou Medical University (Guangzhou Huiai Hospital). All the participants and their guardians provided written informed consent.

In all, 233 subjects in the first year of middle school (age 12–14 yeas) were invited to participate in the trial. Nine subjects refused and 224 participants completed interviews with a psychiatrist and a psychologist to determine eligibility. We used a self-reported, 74-item symptom checklist and a Chinese version of the Bipolar Prodrome Scale-Retrospective: Patient Version (BPSS-R-Pt) to assess current and pass symptoms respectively, based on which research psychiatrists using the DSM-5 criteria to exclude psychiatric disorders. In all, 56 participants with subthreshold mood syndromes were confirmed using the following definition. They either presented (i) subthreshold depression defined as two or more depressive symptoms lasting for at least 1 week but falling short of the criteria for a major depressive episode, or (ii) subthreshold hypomania defined as two or more (up to four if mood was only irritable) manic symptoms lasting at least 4 days or as meeting the hypomanic symptom criteria lasting 2–3 days. The participants of this study were not on any psychiatric medications. Any DSM-4-defined psychiatric disorders were excluded. Other exclusions were physical diseases that were not suitable for running exercise (e.g., cardiovascular and neurological diseases), severe suicidal ideation, mental retardation, and currently having been involved in any formal exercise training programs. All of the participants with subthreshold mood syndromes were invited to do MRI scanning and 39 of 56 (69.6%) completed.

### Randomization and procedures

Randomization code was generated by a specially designed procedure. Randomization was performed in a 1:1 ratio, and the exercise intervention assignment was not masked. But data collectors were told not to ask participant’s study assignment and primary investigators were blinded to allocation. We applied general psychoeducation as a parallel, controlled arm in order to balance potential placebo effect of being treated as it might be seen in the exercise running group. Outcome assessors were instructed not to ask participants’ treatment allocation and were blind to the treatment allocation.

The aerobic exercise group received moderate-intensity running, which was defined as 50–70% of maximum heart rate (calculated as 220-age, beats/minute), 30 min per day, 4 days per week for a total of 3 months. The exercise took place in the school playgroup in the afternoon after class. Each participant’ heart rate was measured with a heart rate monitor right before and after the 30-min running exercise. Because all the participants were in a similar age, a volunteer lead participant in each team that is consisted of ~30 participants helped to meet the exercise intensity (50–70% of maximum heart rate) by adjusting his/her speed. The whole exercise process was video-recorded each time. Approximately 88% participants in the exercise intervention arm finished >80% of the exercise sections.

The control group received a namely psychoeducation treatment, which consisted of three sections of general psychological education, one section of group game, poetry reading, and singing. Approximately 90% participants attended four or more out of six sections.

### Primary outcome measures

The co-primary outcomes were gray matter (GM) volumes and cortical thickness of the ACC (i.e., rostral and caudal).

### Structural imaging data acquisition and processing

Structural imaging data were acquired using a Philips Achieva X-series 3.0 T scanner equipped with an eight-channel SENSE head coil. The scan parameters were described as below: the parameters were: repetition time (TR) = 8.2 ms; echo time (TE) = 3.7 ms; FOV = 256× 256 mm^2^; voxel sizes = 1 × 1 × 1 mm^3^; matrix size = 256 × 256; slices = 188; slices thickness =1 mm; slices gaps = 0 mm.

### MRI data preprocessing and analysis

A quality check for motion and dropout artifacts was conducted before the structural MRI data were preprocessed. Structural MRI data including volumetric segmentation were preprocessed using FreeSurfer software (FreeSurfer 4.0.5, http://surfer.nmr.mgh.harvard.edu). The following preprocessing steps were conducted to estimate the GM in cortical and subcortical areas: motion correction, non-parametric non-uniform intensity correction, transformation of the original volume to the MNI305 atlas using the MINC program MRITOTAL, intensity normalization, skull stripping, automatic subcortical segmentation, GCA atlas registration, removal of neck region, EM registration with the skull, CA labeling (labeling of subcortical structures using the GCA model), white matter segmentation, and cutting of the mid- brain from the cerebrum, and the hemispheres from each other. No manual corrections of the automaged outputs from FreeSurfer were performed. Upon completion of all preprocessing, analyses of the primary ROIs were conducted using SPSS 24 (Chicago, IL, USA).

### Statistical analysis

Participants’ demographic and clinical variables were analyzed using independent-sample *t* test or Chi-square test. Research of interest (ROI) analyses were performed for the ACC GM volume and cortical thickness, including rostral ACC and caudal ACC. The ROI GM volumes and cortical thickness across Time (baseline and follow-up) and Group (exercise intervention group and control group) as well as Group × Time interaction effect were investigated. By testing the Group × Time interaction effect, we tested whether there were neural changes across time, which interacted with the group effect. Specifically, we investigated whether there were significant structural neural changes after the exercise intervention but not the control group. Therefore, the neural correlates were the dependent variables and Group was the between-group independent variable while Time was the within-group independent variable in the present study.

Analyses of the ROI neural correlates were performed using SPSS (Chicago, IL, USA) via employing repeated measure *t* test analysis. Repeated measure *t* test analysis was conducted for the structural neural correlates with Group, Time, and the interaction term of the two as the independent variables. Significance was set based on a two-tailed alpha level of 0.05 for all tests. In all repeated measure *t* test analyses with neural correlates as the dependent variables, the whole-brain volumes were controlled for.

## Results

In all, 223 students were assessed for eligibility, and 56 met the criteria for subthreshold mood syndromes and were randomly assigned to aerobic exercise or psychoeducation controlled. In all, 39 of the 56 participants completed the MRI scan. In total, 20 and 19 subjects were in the exercise and control group, respectively. Baseline demographic information, clinical characteristics, and neural correlates were balanced between the two groups (Table 1). There was no difference in the effect of exercise versus psychoeducation (*p*s > 0.05).

**Table 1:** Demographic and neural correlates of individuals with subthreshold mood syndromes measured at the baseline in exercise intervention and control group in the present study.

	Exercise group (*n* = 21)	Control group (*n* = 18)	Statistics
*Demographics*			
Age (years)	12.67 (0.73)	12.61 (0.50)	*t*_37_ = −0.27, *p* = 0.79
Sex (% males)	38.10	44.44	*X*^2^_1,39_ = 0.69, *p* = 0.75
PHQ-9 at baseline	6.1 (5.0)	3.8 (5.1)	*t*_37_ = 2.00, *p* = 0.17
HCL-15 at baseline	7.7 (2.6)	7.0 (2.2)	*t*_37_ = 0.70, *p* = 0.41
PHQ-9 post-intervention	3.8 (4.3)	3.1 (3.6)	*t*_37_ = 0.281, *p* = 0.6
HCL-15 post-intervention	2.2 (2.5)	1.5 (2.7)	*t*_36_ = 0.049, *p* = 0.825
Whole brain volumes (×1000 mm^3^)	1562.23 (167.79)	1599. 26 (91.55)	*t*_37_ = 0.84, *p* = 0.41
*Neural correlates*			
Rostral anterior cingulate GMV			
Left^a^	29.86 (6.74)	31.55 (4.29)	*t*_37_ = 0.92, *p* = 0.36
Right^a^	21.08 (3.75)	21.95 (4.45)	*t*_37_ = 0.67, *p* = 0.51
Caudal anterior cingulate GMV			
Left^a^	18.17 (4.53)	21.30 (5.65)	*t*_37_ = 1.92, *p* = 0.06
Right^a^	22.04 (5.68)	25.14 (3.57)	*t*_37_ = 2.00, *p* = 0.05
Rostral anterior cingulate CTh			
Left^b^	3.15 (0.27)	3.14 (0.14)	*t*_37_ = −0.18, *p* = 0.86
Right^b^	3.14 (0.22)	3.14 (0.16)	*t*_37_ = −0.05, *p* = 0.96
Caudal anterior cingulate CTh			
Left^b^	2.97 (0.15)	2.99 (0.24)	*t*_37_ = 0.32, *p* = 0.75
Right^b^	2.85 (0.24)	2.85 (0.23)	*t*_37_ = −0.09, *p* = 0.93

Numbers in brackets represent the standard deviation (SD); cortical thickness: CTh; gray matter volumes- GMV. PHQ-9: Patient Health Questionnaire. HCL-15: Hypomania Symptom Checklist: ^a^(100 mm^3^); ^b^(×1 mm); *P < 0.05.

### Exercise effects on the brain structure

**Fig. 1:**
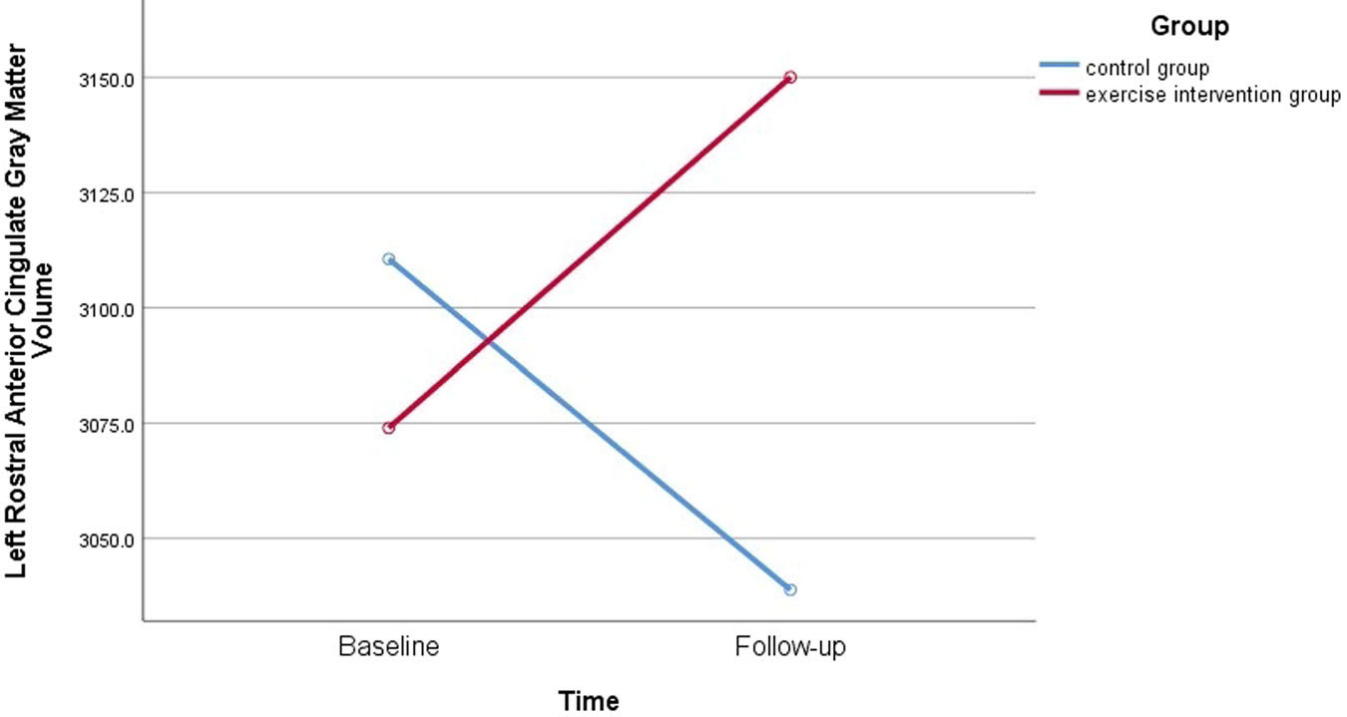
The gray matter volumes of the left rostral anterior cingulate in healthy controls and intervention group at the baseline and follow-up. The Group × Time interaction effect was significant revealing that increased left rostral anterior cingulate gray matter volumes were found in the exercise intervention group.

The repeated- measures *t* test results showed that the Group × Time interaction effect was significant (F_1,30_ = 5.73, *p* = 0.02), whereas the Time (F_1,30_ = 3.75, *p* = 0.06) and Group effect (F_1,30_ = 0.17, *p* = 0.68) were not significant in the left ACC GM volume (Fig. 1). Either Group × Time interaction effects or main effects were not significant in the right rostral ACC GM volume (*p*s > 0.05). For both the left and right caudal ACC GM volume, either Group × Time interaction effects or main effects were not significant (*p*s > 0.05).

**Fig. 2:**
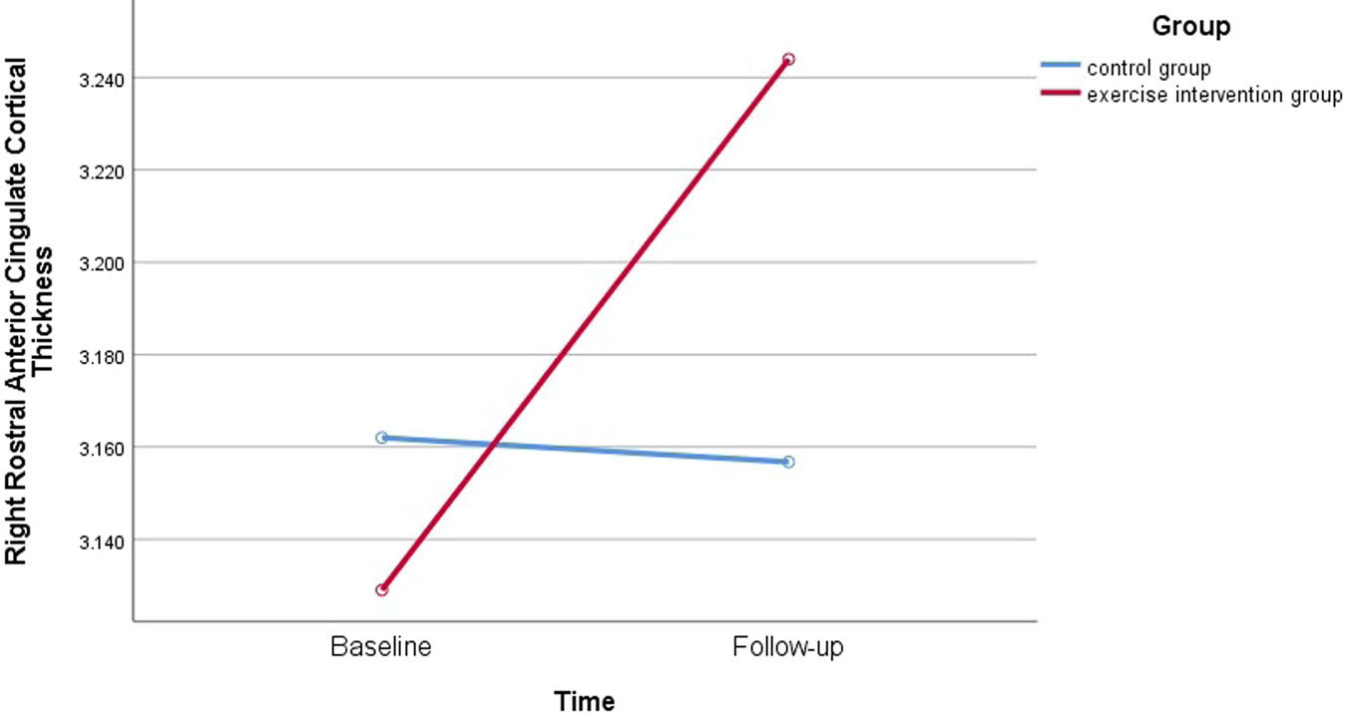
The cortical thickness of the right rostral anterior cingulate in healthy controls and intervention group at the baseline and follow-up. The Group × Time interaction effect was significant revealing that increased right rostral anterior cingulate cortical thickness were found in the exercise intervention group.

As to the right rostral ACC cortical thickness, the Group × Time interaction effect was significant (F_1,30_ = 7.83, *p* = 0.01) (Fig. 2). Although the Time (F_1,30_ = 0.12, *p* = 0.73), Group effect (F_1,30_ = 0.17, *p* = 0.68) were not significant. Either Group × Time interaction effects or main effects were not significant in the left rostral ACC cortical thickness (*p*s > 0.05). For both left and right caudal ACC cortical thickness, either Group × Time interaction effects or main effects were not significant (*p*s > 0.05).

## Discussion

To the best of our knowledge, this is the first RCT to investigate structural brain changes in adolescents with subthreshold mood disorders. In our study, we identify potential changes in key brain areas implicated in mood disorders, which may improve in response to 12-week moderate-intensity aerobic exercise, i.e., GM volume of the left rostral ACC and cortical thickness of the right rostral ACC.

Previous literature has confirmed that exercise can help for subthreshold and established mood disorders, yet the mechanisms are poorly understood^23^. The ACC investigated in this study have been identified as being impacted and reduced in adolescent with subthreshold depression and hypomania^13,29^. Relatively mild mood fluctuations in the forms of subthreshold depression and hypomania particularly during adolescence are core features of disturbances in mood regulation^30,31^. Our sample comprised of adolescents manifesting both subthreshold depression and hypomania. Despite concrete mood symptoms, the IMAGEN study showed that both adolescents with subthreshold depression and bipolar symptoms had decreased structural volumes in the ACC^32,33^. ACC is postulated as a hub in the brain networks^34,35^ and implicated in neurocognitive function and reward learning and decision-making^36^ in addition to conflict monitoring^37^ and social processing^38^. Moreover, adolescents with subthreshold mood syndromes are at higher risk of developing mood disorders^30,39^. Adolescents with depression had decreased negative connectivity between the rostral ACC and the bilateral amygdala, suggesting deficient control of the rostral ACC over amygdala^40^. Furthermore, the top–down control of ACC over the amygdala has been suggested to be an important feature of pathopsychology in bipolar disorder^28^.

Patients with bipolar disorderat the very early illness course were also found to be have decreased structural volume of ACC^41,42^. Its metabolic changes were reported prior to depression^43^. We previously reported that individuals with subthreshold mood syndromes and a family history of bipolar disorder displayed a disregulated correlation between rostral ACC and the level of IL-6 and attention function^44^ when compared with those without symptoms.

Clinical trials of exercise (and increased physical activity) that have investigated the exercise effects on the ACC were limited and are mainly on elderly individuals with or without cognitive impairments^24,45^. For instance, Ruscheweyh et al., assigned a group of 62 elderly healthy individuals into medium-intensity exercise (nordic walking, *n* = 21), low-intensity exercise (gymnastics, *n* = 20) or no intervention (control, *n* = 20). The two exercise groups were asked to exercise for 50 mins, three times per week for a total of 6 months. They found that the changes in the GM volume of the left ACC was correlated with physical activity^24^. A clinical study of Baduanjin—a type of mind-body exercise—found that Baduanjin increased activity in the ACC at rest in patients with mild cognitive impairment^45^. Another clinical trial on older individuals (average age = 70) also found that 6 months of progressive resistance physical training could increase GM volume in the posterior ACC^46^. Given that increasing evidence showing individuals with subthreshold depression and hypomania and patients with bipolar disorder displayed decreased ACC structure compared with the health individuals, and that exercise can help ameliorate depressive symptoms in adolescents^47^, we speculate that the increased GM volume of the ACC might be beneficial to individuals with subthreshold mood symptoms. Nonetheless, we did not find that the changes of ACC were positively associated with that of clinical symptoms and neurocognition. Given the role of ACC as a key region in mood regulation, future study needs to investigate whether the change of ACC including structural and functional change could impact other behavioral measures related to mood regulation so as to elucidate the mechanism of exercise-induced change of the ACC in individuals with subthreshold mood symptoms.

It is worth noting that we did not find any exercise effect on the clinical symptoms (e.g., PHQ-9) and neurocognitive function (measured by the MATRICS Consensus Cognitive Battery, MCCB) (the results owing to be reported elsewhere). The changes of the ACC structure were not correlated with that of clinical symptoms and of neurocognition (data not shown). It is possible that the changes in the rostral ACC may not be sufficient to cause measurable behavioral or symptom changes. Moreover, as these individuals’ symptoms (including depressive and hypomanic) and cognition were not severely impaired at baseline, the sample size of our trial may not have enough statistical power to detect clinically meaningful difference.

### Strengths and limitations

This exercise trial was well conducted in the school playground and video-recorded. The exercise protocol including exercise intensity and duration was strictly followed. Participants had high attendance. Although there were some limitations that should be noted when interpreting our findings. First, the sample size was relatively small (*n* = 39). As a consequence, it may not have the statistical power to detect exercise effects on the behavioral measures, which could help appreciate clinical implications of the enhanced structural changes identified. Second, we only included adolescents aged between 12 and 14 years and the finding may not be applied to older adolescents. Third, the trial only applied running exercise. It is not clear whether the exercise effect on the ACC structure could be generalized to other types of exercise. Future study needs to compare the effects of different types of exercise on the brain structure. Fourth, the exercise trial was short-term. It is not clear whether the structural changes identified could affect the adolescents’ long-term outcomes. Finally, in this 6-week trial, we did not follow-up those high-risk adolescents for developing mood disorders. It is of great interest to the field whether exercise intervention can delay or prevent the onset of mood disorders. Future study need to answer this scientific question.

In conclusion, our data show that 12-week, moderate-intensity aerobic exercise can positively change the rostral ACC structure in adolescents with subthreshold mood syndromes. Given the ACC has a key role in a array of critical functions and its reduced structure is seen at the very early course of mood disorder, our findings support implementation of aerobic exercise in this high-risk population.
